# NORMA-Gene: A simple and robust method for qPCR normalization based on target gene data

**DOI:** 10.1186/1471-2105-12-250

**Published:** 2011-06-21

**Authors:** Lars-Henrik Heckmann, Peter B Sørensen, Paul Henning Krogh, Jesper G Sørensen

**Affiliations:** 1 National Environmental Research Institute, Aarhus University, Department of Terrestrial Ecology, Vejlsøvej 25, DK-8600 Silkeborg, Denmark

## Abstract

**Background:**

Normalization of target gene expression, measured by real-time quantitative PCR (qPCR), is a requirement for reducing experimental bias and thereby improving data quality. The currently used normalization approach is based on using one or more reference genes. Yet, this approach extends the experimental work load and suffers from assumptions that may be difficult to meet and to validate.

**Results:**

We developed a data driven normalization algorithm (NORMA-Gene). An analysis of the performance of NORMA-Gene compared to reference gene normalization on artificially generated data-sets showed that the NORMA-Gene normalization yielded more precise results under a large range of parameters tested. Furthermore, when tested on three very different real qPCR data-sets NORMA-Gene was shown to be best at reducing variance due to experimental bias in all three data-sets compared to normalization based on the use of reference gene(s).

**Conclusions:**

Here we present the NORMA-Gene algorithm that is applicable to all biological and biomedical qPCR studies, especially those that are based on a limited number of assayed genes. The method is based on a data-driven normalization and is useful for as little as five target genes comprising the data-set. NORMA-Gene does not require the identification and validation of reference genes allowing researchers to focus their efforts on studying target genes of biological relevance.

## Background

Real-time quantitative PCR (qPCR) represents the current state-of-the-art approach for measuring gene expression; and the method has numerous applications in both biology and biomedicine. Although qPCR is a robust technique results can vary depending on factors such as RNA integrity, reverse transcriptase (RT) efficiencies, sample-to-sample variations in amplification efficiency, and variation in cDNA sample loading. Using equal sample sizes, assessing RNA integrity and equalizing RNA concentrations prior to RT are fundamental normalization steps in qPCR [1]. Still, normalization to some internal control is essential for accurate qPCR in order to balance sample-to-sample variations within the RT and PCR reactions. Currently, the preferred internal control is achieved by using reference genes (also referred to as housekeeping genes) or better a normalization factor based on several reference genes calculated using e.g. geNorm [2]. However, the use of reference genes suffer from a circular argument; i.e. we normalize target gene expression data to exclude the systematic variation by the means of reference gene expression data obtained by the same method as the data that need normalization. Thus, an assumption for using reference genes is that they are unaffected by the experimental treatment(s) and measured accurately and without error, as we rely on the target gene data to be correctly normalized by the reference gene(s). The circularity is partly evened out by the normalization factor approach, based on the expression of several reference genes, following the assumption that the distribution of three or more reference genes is more accurately estimating systematic error than the distribution of only one gene [2]. However, in many studies reference genes are chosen more or less randomly and are not always being validated for the particular experimental conditions. A further downside is that it can be difficult to find suitable reference genes for certain experimental conditions that affect gene expression broadly [3]. Searching for and validating reference genes is thus both time and money consuming and might not always be practical or successful. Heterogeneous samples, however, require a robust normalization strategy. Yet, conventional normalization may introduce unintentional random changes to the variance and mean expression of target genes in lack of good reference genes. This may cause invalid conclusions, and prevent good target gene data-sets from being accurately analyzed increasing the risk of making type I and II statistical errors. Thus, the use of reference genes has become the chosen method not because it is extremely good, but because it represents the best available option.

Here we present an algorithm, NORMA-Gene, which is applicable to all biological and biomedical studies, especially those that are based on a limited number of genes measured with qPCR. The method is based on a data-driven normalization of target genes and is valid for as little as five target genes comprising the data-set. It does not require the use of reference genes allowing researchers to focus their efforts on studying target genes of biological relevance.

## Methods

We have defined two levels of variance in the qPCR data-sets. The first level refers to among replicate variation (hereafter referred to as *bias*). This variation includes biological variation and variation in RNA extraction and reverse transcription (RT) efficiency. The second level of variance refers to the variation among the measured genes within a single replicate (hereafter referred to as *variation*). This variance reflects the technical and random variation in the qPCR part of the procedure. NORMA-Gene reduces systematic and artificial between-replicate bias utilizing the entire data-set of the target genes being studied. The approach is based on calculating mean expression values for each replicate across the studied target genes and subsequently estimating a normalization factor that estimates and reduces the systematic bias of a replicate across all genes. For this a Least Square method is applied to secure the best possible minimization of variability in the data-set based on the bias between replicates within treatment. Least square regression is non-robust to outliers and careful quality control measurement throughout the qPCR process (i.e. verifying equal PCR efficiencies, verifying PCR product quality by melting curve inspection, etc.) is thus essential pre-normalization. The procedure allows the estimation of the remaining variance in the data-set, not explained by experimental bias between the replicates, and the calculation of the precision (variance) of the identified bias. The procedure is only affected by the number of replicates and genes within a treatment; however, for more than three replicates the number of genes is predominantly of importance for the precision of the normalization. The procedure is not affected by up-or down-regulation as no between treatment relations are being used. Detailed derivation of the following equations is available in Additional file 1, Appendix A.

### The mathematical theory behind NORMA-Gene

The NORMA-Gene algorithm for a single treatment is shown below and in more detail in Additional file 1, Appendix A. Within each treatment of the non-normalized data-set, *n *genes are measured for *m *replicates. The normalization will take place using log-transformed data. The normalization factor for each replicate within a treatment is calculated as:(1)

where *a_j _*is the bias coefficient for replicate *j*, *i *is the index for the genes and *N_j _*is the number of genes that are recorded for replicate *j*,  is the measured gene expression value for sample *j *and gene *i*, and  is the estimated mean value for log-transformed gene *i *data

The standard deviation of *a_j _*is found as a global standard deviation within the same treatment assuming that all *a_j _*are following the same probability distribution function. A first order uncertainty assessment for the estimation of *a_j _*is given as:(2)

where *σ*_log*X *_is the standard deviation of *a_j _*assuming variance homogeneity which is similar to assuming the same relative (percentage) error for the data:(3)

Eq. 2 will tend to take a simple approximately form: *a_j _*will tend to be close to unity otherwise the data errors are very large and *M_i _*will normally be more than 3 and thus the product of *N_j _*·*M_i _*will tend to be much larger than *N_j _*and thus the ratio  will dominate in Eq. 2 (and see Additional file 1, Appendix A for a detailed derivation of the equations). If there are not very many missing data then *N_j _*will tend to be constant and equal *n *and if all these statements are correct then Eq. 2 can be reduced to:(4)

### Data handling

Target gene normalization has been simplified by automating all calculations in an Excel workbook (Microsoft) entitled NORMA-Gene (freely available upon request from the corresponding author. This macro-based workbook enables swift normalization of imported raw expression data. It is feasible to use both fluorescent data derived from the software available on the used qPCR platform, or computed by e.g. the DART-PCR algorithm [4].

### Missing data

In even the most carefully designed and executed experiment missing data might occur. This is problematic as many qPCR studies are performed with limited sample sizes. Especially, the occurrence of missing a replicate of the reference gene is problematic as it leads to the loss of an entire target gene (biological) replicate when relying on reference gene normalization. NORMA-Gene is very flexible and little affected by missing data, and is able to normalize samples in treatments with missing data. The calculated NORMA-Gene normalization factor is valid for and can be used on all genes in a corresponding replicate, as the factor is replicate-specific.

**Figure 1:**
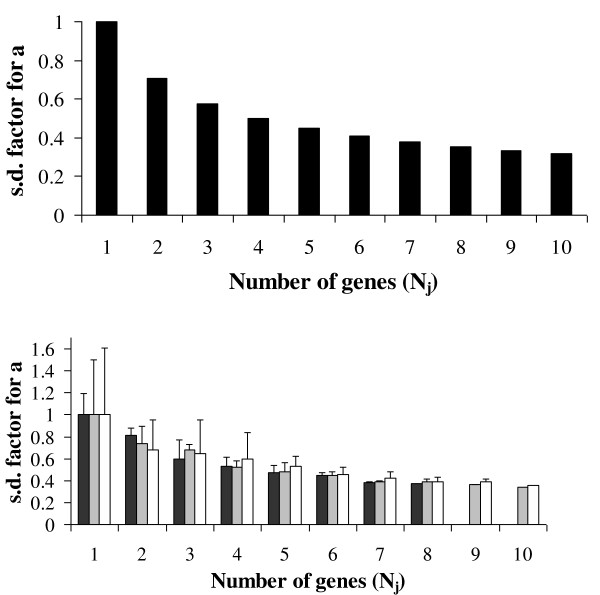
**The figure shows the theoretical (top panel) and empirical (bottom panel) relative variance reduction with number of genes used for NORMA-Gene normalization**. As the number of genes increases the relative standard deviation for the fitted ***a*** is reduced as displayed (see Eq. A8 in Additional file 1, Appendix A for the mathematical rationale). Top panel: The figure shows the theoretical prediction that the standard deviation of the fitted ***a*** is more than halved when using five genes. Adding further genes to the analysis only slightly improves the estimate of ***a***. Bottom panel: The figure shows the reduction in the standard deviation of the fitted ***a ***when NORMA-Gene is applied to real data. Dark gray, light gray and white bars represent data-sets I, II and III, respectively. As the improvements (reduction) of the standard deviation is a result of adding one more gene to the analysis, the result is dependent on the genes included in each data-set when all genes are not used. Thus, means and error bars represent three different randomly normalizations. These corroborate with the theoretical predictions of stable and robust normalization when five or more genes are used.

The NORMA-Gene normalization improves gradually with the number of genes that are included in the target gene data-set (see Figure 1 and results). Thus, as long as a minimum number of data points (five or more) is available within a replicate (across genes) normalization by NORMA-Gene can be performed. It is not required that the same five data points are available in all replicates within a treatment, and as such NORMA-Gene can proceed even with quite extensive missing data.

### Benchmarking NORMA-Gene on artificial data-sets

As the true values of real data-sets are always unknown it can be difficult to evaluate normalization procedures. Thus, we generated artificial data-sets to evaluate the performance of reference gene and NORMA-Gene normalization. Each data-set was comprised of a single treatment with four replicates, with one reference factor (which could represent a single reference gene or a normalization factor based on several genes) and eight target genes measured in each replicate. The artificial data-sets were generated in two steps, each with independent variation. First, we sampled four replicates from a treatment with a true mean of zero and an experiment dependent bias (among replicate variation, see above). The second step was to generate the variation (among genes within a replicate). The targets genes were in all cases sampled with a fixed variation of 10% while the reference factor was sampled with different levels of variation. By comparing normalized data to the known true means we were able to evaluate and validate the performance of the NORMA-Gene normalization relative to the reference gene method at different bias-to-variation ratios and at different variation in reference gene-to-variation in target gene ratios. Note that the variation referred to here does not regard the among treatment variation in the reference gene or normalization factor as is usually the concern of this type of normalization. In the artificial data we assumed no between treatment effects and the reference gene or normalization factor is thus assumed to reflect the optimal situation with no variation among treatments. Rather the sampled variation of both reference and target genes only reflect the precision by which the signal (gene expression) is measured. Each set of experimental conditions were re-sampled 40 times.

### Data analysis

Differences in the efficiency of reducing variances by the two normalization methods (reference gene(s) v NORMA-Gene) were tested with Wilcoxon's signed-ranked test for two groups of paired observations [5].

## Results

**Figure 2:**
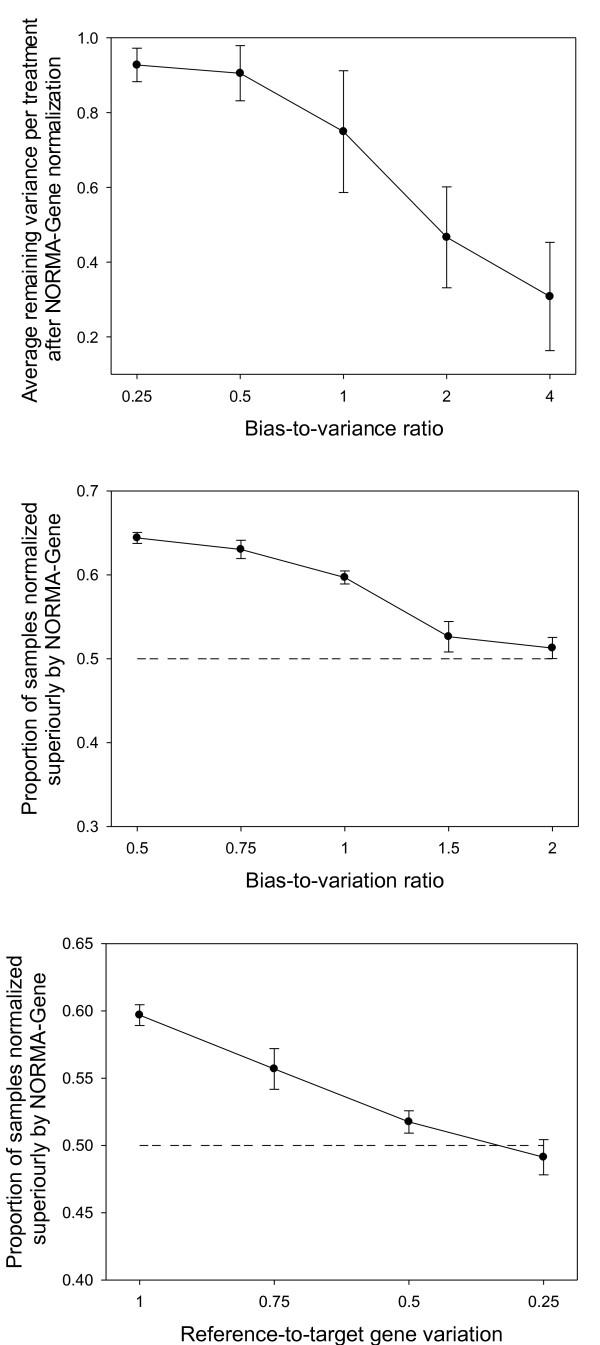
**The figure shows the results of analysis of artificial data-sets**. Throughout means ± SEM of the 40 re-samplings are given. Top panel: This panel shows the variance reduction by normalization and how this depends on the bias-to-variation ratio. Increasing the bias-to-variation ratio in the artificial data leads to more variance being removed from the data-set by NORMA-Gene. Middle and bottom panels: These panels show the effectiveness of NORMA-Gene versus reference gene normalization and the dependence of different parameters. For each of the re-samplings (see methods for detailed information on the construction of the data-sets) the proportion of NORMA-Gene normalized data points that were closer to the true mean was calculated and is shown on the y-axis (0.5 represent equal performance of NORMA-Gene and reference gene normalization). Middle panel: Here the x-axis represents the ratio between bias (between replicate variation) and variation (between genes within replicate variation). As bias among replicates increase the performance of NORMA-Gene decreases. No qualitatively difference was observed in the performance of NORMA-Gene between re-sampling from eight or four genes. Bottom panel: The x-axis represents the ratio of reference gene-to-target gene variation (here 1 represent equal variation and 0.25 represent four-fold decreased variation in the reference factor).

The results of the analysis of artificial data-sets are shown in Figure 2. To evaluate the performance of the NORMA-Gene normalization on the artificial data we tested; i) the variance reduction at different bias-to-variation ratios using NORMA-Gene. Further, to test the effects of normalization methods we compared performance on two sets of parameter combinations in artificial data; ii) the bias-to-variation ratio, and iii) the ratio of reference-gene variation relative to target-gene variation. In these two latter tests the quality of the normalization was judged by the deviation of expression level in normalized data from the true expression values.

Increasing the bias-to-variation ratio in the artificial data leads to more variation being removed from the data-set by NORMA-Gene (Figure 2, top panel). This is not surprising as NORMA-Gene is designed to identify and reduce bias, and as bias increases as a larger proportion of the total variance is being removed. The relationship was little affected by number of replicates (2-4) and only slightly by number of genes (3-12 was tested, but only 3 genes showed a difference from the situation with other genes).

The result of the second test showed a superior performance of the NORMA-Gene method with a larger degree of samples closer to the true mean than when reference gene normalized under a large range of conditions (Figure 2, middle panel; Additional file 2, Figure S1). The bias (among replicate variation) to variation (among genes within replicates) ratio affected the relative performance of the NORMA-Gene normalization method. With increasing bias-to-variation ratio, the performance of NORMA-Gene decreased until the two methods were equal with the bias being 2 times larger than the variation; and the reference factor method was only superior at bias-variation ratios larger than 2. Furthermore, re-sampling the data-set with only four genes did not affect the performance of the NORMA-Gene methods confirming the stability of the method even with few genes measured (not shown). As the bias-to-variation ratio affects the effectiveness of NORMA-Gene compared to reference gene normalization it is highly relevant to estimate this ratio in real data-sets before using the NORMA-Gene procedure. However, the bias-to-variation ratio in real data-sets is difficult to estimate. One way is to use the relationship between bias-to-variation ratio and average variance reduction as seen in Figure 2, top panel. Interestingly, the average variance reduction in the three real data-sets (0.78, 0.78 and 0.91) suggests that the bias-to-variation in these data is between 1.0 and 0.5 and thus in a range where NORMA-Gene is performing better than reference gene normalization.

The third and final test on artificial data concerned the level of sample variation when sampling a reference gene/reference factor. To remind, this level of variance is the sampling variation among genes from the same sample (replicate) and not among treatment variance. Thus, for the validation of the method using artificial data we have assumed the perfect reference gene, i.e. no variation among treatments (the reference genes are all sampled from a distribution with the exact same mean). This is the optimal situation and quite unrealistic to achieve in many studies as argued in the text. The variation we allowed to vary is the technically variation, which determines how well the qPCR procedure measures the true amount of cDNA in the sample. The realistic value in this case most be 1 (i.e. equal variation in reference and target genes), as reference genes and target genes are measured in the same samples having gone through the same extraction and cDNA synthesis procedure, unless more replicates is used for reference genes, or more care is taken by the experimenter while measuring these genes compared to when measuring target genes. Thus, by applying "perfect" reference genes and allowing these to be measured more precisely than target genes we are being very conservative in the test of the NORMA-Gene procedure. Here we found, as expected, that increasing the precision of the reference gene/reference factor increased the performance of this normalization method compared to NORMA-Gene (Figure 2, bottom panel; Additional file 2, Figure S1). The reference gene/reference factor approach needs to be measured four times as precise as the target genes to result in a normalization efficiency equal to the NORMA-Gene method. As discussed above this will be difficult to achieve using a single reference gene, but possible using a normalization factor based on multiple genes. Note that any effect of treatment on reference genes will add to the level of noise and decrease the precision of reference factor normalization; rendering this situation of four times precision yielding equal normalization a conservative estimate. In experiments using harsh treatments, e.g. stress research, those reference genes, totally unaffected by treatment, might prove very difficult to identify.

### Validation on real data-sets

We applied the NORMA-Gene algorithm to three different data-sets (see Additional file 3, Table S1 for an overview of data-set I-III): I) A data-set on springtails with six target and one valid reference gene [3]; II) a data-set on earthworms with nine target and one valid reference gene (unpublished data-set); and III) a data-set on *Daphnia *with 10 target and a geNorm based normalization factor using three valid reference genes [6]. In all three data-sets the reported results are for target genes only, but reference genes were included in the NORMA-Gene algorithm for data-set I and II, to improve the stability of the normalization (see below).

The NORMA-Gene algorithm uses a Least Square method to secure the best possible minimization of variability in the data-set due to bias between replicates (see Methods). The algorithm predicts the relative reduction in variation as a function of the number of genes comprising the data-set. NORMA-Gene generates a stable normalization with as little as five genes, and addition of further genes to the data-set has little relative effect on the normalization output (Figure 1). The predicted effect of the number of genes on the normalization output was confirmed in all three data-sets (see Additional file 4, Figure S2) verifying that using a data-set of five or more genes is a conservative approach for obtaining a stable normalization.

Validation of the normalization efficiency was achieved by looking at the distribution of standard deviations for each sample in the raw data after NORMA-Gene normalization, and after normalization to a reference gene (data-sets I and II) or a normalization factor based on three reference genes (data-set III). We plotted the variance reducing effect of reference gene or NORMA-Gene normalization on variances relative to the variance of raw data (Additional file 5, Figure S3). The variance reduction was significantly larger following NORMA-Gene normalization for all three data-sets (Additional file 5: Figure S3A, t_s _= 7.6, P < 0.001; Figure S3B, t_s _= 6.6, P < 0.001; Figure S3C, t_s _= 2.8, P < 0.01); thus significantly reducing the variance compared to the reference gene(s) based normalization.

The relative effect of the different normalization approaches on mean expression values revealed that traditional reference gene normalization affects mean expression values to a greater extend than NORMA-Gene normalized data (see Additional file 6, Figure S4). A comparable effect on mean was seen in the artificial data when considering a bias-to-variation ratio of real data-sets of between 0.5 and 1.0 (see Additional file 2, Figure S1). The mathematics behind NORMA-Gene ensure that mean expression values within treatment are affected minimally by normalization, and thus deviate as little as possible from the raw expression values (see discussion on effects on mean expression values).

## Discussion

Within microarray analysis a data-driven normalization, e.g. global LOWESS, has long been applied [7], but these methodologies rely on very large data-sets in order to deliver meaningful outputs. Recently, two qPCR-based methods using data-driven normalization have been published providing proof of principle. The first describes a microarray inspired data analysis approach where quantiles are applied to normalize the target genes; yet the approach is only applicable to high-throughput qPCR analysis involving 50 to several thousand genes [8]. The other method is developed for normalization of microRNAs quantified using qPCR [9] and is according to the authors only applicable to large-scale gene expression studies. However, no data-driven normalization alternative is currently available for small-scale mRNA expression studies using qPCR. We show here that our method produces normalized data which are closer to the true means under a range of realistic variance parameters. Furthermore, we show that NORMA-Gene produces reduced experimental bias (within treatment variation) to a higher extend and thus outperforms the current approach based on reference genes; even when good reference genes are available as in the case of data-set III [6,10]. We furthermore show that a reliable normalization may be obtained with as little as a five genes comprising the data-set and verify this on the artificial data-sets. The results of normalization and the distributions of the normalized data show high comparability between artificial and real data suggesting that the artificial data are behaving in a similar manner to real data and thus that the results of artificial data can be taken as relevant and meaningful for the validation of the method.

NORMA-Gene is designed to reduce within treatment variation only and thus has little effect on the mean expression values between treatments. Contrary, reference gene(s) normalization can have a large effect on the estimated mean expression values across treatments as this approach affects both within and between treatment variation. This is not a problem if the reference gene(s) have been measured correctly, and thus adjust the raw data accurately. However, our analysis of the artificial data-sets show that a four-fold reduction in variation in the used reference factor is needed to obtain similarly good results with reference genes. Thus, reference genes, being measured with the same technique as target genes, will realistically be measured with similar error as target genes across treatments; or are affected by the treatment (which is ignored in the artificial data-sets to produce conservative estimates). Normalization factors based on more than one reference genes, i.e. using the geNorm procedure, reduces this problem but does not eliminate it as seen for our real data-set III. This will introduce variation across treatments, which was not present in the original data. Furthermore, biological variation may distort the "true" mean expression values when non-validated reference genes are being used for normalization. In our opinion, the measured raw data provide the best estimate of mean expression values available; and only minor changes to these means following normalization are to be expected as achieved by NORMA-Gene normalization.

NORMA-Gene normalization is virtually non-affected by the above-mentioned issues regarding reference gene related effects on mean expression values. However, NORMA-Gene requires using a block design to minimize variation across treatments. If the experimental setup is based on a block design and any handling (i.e. RNA extraction, cDNA synthesis) of the samples has been conducted block-wise, between treatments variation will be absolute minimal. Hence, a block design is recommended regardless of which type of normalization that is being applied to the raw data.

## Conclusions

The NORMA-Gene algorithm is applicable to small data-sets allowing more target genes to be investigated. Unless reference gene(s) are un-affected by treatment, can be measured much more precisely than target genes or data-sets has bias surpassing variation by more than 2 fold (i.e. huge technical variation) the NORMA-Gene algorithm produces equal or better normalization than reference gene(s). Thus, no reference gene(s) are needed when five or more target genes are being analyzed (i.e. focusing of resources); although it could be an advantage to include a number of valid reference genes to serve as "negative biological controls". Good quality (random biased) data are achieved when using NORMA-Gene; and the data are less sensitive to experimental and biological outliers than the current approach based on reference genes. The statistical assumption of the method is that a relative sample error is randomly distributed across the mean of all genes and replicates within treatment. This assumption is more likely to be met than the assumptions associated with reference gene normalization.

The NORMA-Gene approach requires applying a block design, i.e. carefully processing the same replicates from each treatment together. Under these circumstances the design will control for all experimental variability between treatments, leaving NORMA-Gene to normalize the within treatment variation. Applying a block design has the additional advantage of minimizing any potential confounding problems associated with co-regulated genes (e.g. originating from the same pathway) that might occur when using reference genes. NORMA-Gene utilizes a general statistical normalization approach and may thus also be applied on other similar molecular data-sets (e.g. proteomics and metabolomics).

## Competing interests

The authors declare that they have no competing interests.

## Abbreviations

*a_j_*: bias coefficient for replicate *j*; *i*: index for the genes; *N_j_*: number of genes that are recorded for replicate *j*; *m*: number of replicates; *M_i_*: number of replicates for which data exist for gene *i*; : measured gene expression value for sample *j *and gene *i*; : estimated mean value for gene *i*; RT: reverse transcriptase; qPCR: quantitative PCR.

## Authors' contributions

LHH and JGS developed the idea and the first version of the NORMA-Gene algorithm, validated its performance and drafted the manuscript. PBS refined the algorithm and developed the macro-based NORMA-Gene Excel workbook. PHK provided statistical input. All authors read, contributed intellectually and approved the final manuscript.

## Supplementary Material

Additional file 1 **Appendix A**. The file is an appendix providing detailed derivation of the equations underlying the NORMA-Gene algorithm. Click here for file

Additional file 2 **Figure S1**. The figure shows scatter of the normalized mean expression values in artificial data (see summary of this statistics in Figure 2). Click here for file

Additional file 3 **Table S1-Overview of analyzed data-sets**. The table displays a table of experimental information related to the analysed data-sets (I-III). Click here for file

Additional file 4 **Figure S2**. The figure provides a validation of the effect of number of genes on mean and standard deviation of the fitted *a *for the real data-sets. Click here for file

Additional file 5 **Figure S3**. The figure shows the relative effect of normalization on expression values in each replicate for the real data-sets. Click here for file

Additional file 6 **Figure S4**. The figure shows the relative effect of normalization on variation in each treatment for the real data-sets. Click here for file
