# Supplementary material for: NORMA-Gene: A simple and robust method for qPCR normalization based on target gene data
Source: BMC Bioinformatics. 2011 Jun 21;12:250. doi: 10.1186/1471-2105-12-250 (PMC3223928; doi:10.1186/1471-2105-12-250)
Supplement: Additional file 1 — Appendix A. The file is an appendix providing detailed derivation of the equations underlying the NORMA-Gene algorithm. [file 1471-2105-12-250-S1.DOC]

**Additional file 1: Appendix A**

The following equations are defined for one treatment, assuming that the error is proportional to the value of the measured gene expression yielding a log-normal distribution that is common for all genes. It is also attractive to apply log-normal statistics to the data, due to the non-normal behaviour for larger corrections. The reason for this is that the signals are always positive and thus the normal distribution will become truncated if the standard deviation (SD) is not very much smaller than the mean. The log-normal distribution approximates closely a normal distribution for small SD and reproduces normal statistics under such circumstances; see more about the application of log-normal distributions in [1].

The mathematical problem is to apply the relation $a_{j}\cdot X_{j}^{i}$ to minimize the variance around the mean value of al replicates for gene *i*. For logarithmic transformed data this means: $\log(a_{j}\cdot X_{j}^{i})=\log(a_{j})+\log(X_{j}^{i})$, where log() is applied as the natural logarithm in the supporting excel sheet. A least square approach was selected to identify the value of $\log(a_{j})$based on the data set:

(A1)

Where

$\overline{\left(\log X\right)_{i}}=\frac{1}{M_{i}}\cdot\sum_{j=1}^{j=M_{i}} \log(X_{i}^{j})$ (A2)

$N_{j}$is the number of data points (genes) of which gene expression is measured for replicate *j* and *M_i_* is the number of data points of which gene expression is measured for gene *i*. For a data set with no missing data (replicates and/or genes) $N_{j}$= *n* and $M_{i}$ = *m*. If there are missing data in the data set then $N_{j}$will not have the same value for all values of *j* (replicates) and $M_{i}$will not be similar for all values of *i* (genes).

Differentiating Eq. A1 with respect to $\log(a_{j})$ yields:

$\frac{\partial O_{j}}{\partial\log(a_{j})}=\sum_{i=1}^{i=N_{j}} \left[2\log(a_{j})+2\log(X_{j}^{i})-2\cdot\overline{\left(\log X\right)_{i}}\right]$ (A3)

The minimal variance exist for the value of $a_{j}$ that satisfy: $\frac{\partial O_{j}}{\partial a_{j}}=0$ and the solution for this problem is

$\log(a_{j})=\frac{\sum_{i=1}^{i=N_{j}} \left[\overline{\left(\log X\right)_{i}}-\log(X_{j}^{i})\right]}{N_{j}}$ (A4)

The variation of $a_{j}$caused by variation in the data can be estimated using a first order Taylor approximation:

$\sigma_{\log(á)}^{2}\approx\sum_{i=1}^{i=N_{j}} \left[\sigma_{\log(X)}^{2}\cdot\left(\frac{\partial\log(a)}{\partial\log(X)}\right)^{2}\right]$ (A5)

The first order Taylor approximation is based on the assumption that the gradient of log($a_{j}$) is constant for all possible values of log($a_{j}$). The gradient is found by differentiating Eqs. A4 and A2:

$\frac{\partial\log(a)}{\partial\log(X)}=\frac{1}{N_{j}\cdot M_{i}}-\frac{1}{N_{j}}$ (A6)

Combining Eqs. A5 and A6 and assuming $\sigma_{\log(X)}^{2}$constant yields:

$\sigma_{\log(a)_{j}}\approx\sigma_{\log(X)}\cdot\sqrt{\sum_{i=1}^{i=N_{j}} \left[\frac{1}{N_{j}\cdot M_{i}}-\frac{1}{N_{j}}\right]^{2}}$ (A7)

Where the standard deviation of log(*X*) is estimated as

$\sigma_{\log(X)_{j}}=\sqrt{\frac{1}{n}\sum_{i=1}^{i=n} \left[\frac{1}{\left(M_{i}-1\right)}\sum_{j=1}^{j=M_{i}} \left(\log(X_{i}^{j})-\overline{\left(\log X\right)_{i}}\right)^{2}\right]}$ (A8)

This equation assumes variance homogeneity between the genes in the log transformed data and an first order Taylor approximation is used to estimate the standard deviation of *a* based on the standard deviation of log(*a*) as:

$\sigma_{a_{j}}\approx\sigma_{\log(a)}\cdot\frac{da}{d\log(a)} , \frac{da}{d\log(a)}=a$ (A9)

Combining Eqs. A7 and A9 yields:

$\sigma_{a}\approx\sigma_{\log(X)}\cdot a_{j}\cdot\sqrt{\sum_{i=1}^{i=N_{j}} \left[\frac{1}{N_{j}\cdot M_{i}}-\frac{1}{N_{j}}\right]^{2}}$ (A10)

This equation will tend to take a simple form based on the following arguments: *a_j_* will tend to be close to unity otherwise the data errors are very large and *M_i_* will normally be more than 3 and thus the product of $N_{j}\cdot M_{i}$ will tend to be much larger than $N_{j}$ and thus the ratio $\frac{1}{N_{j}}$ will dominate in Eq A10. In most cases $N_{j}$ will tend to be constant and equal *n* and if all these statements are combined then Eq A10 becomes:

$\sigma_{a}\approx\frac{\sigma_{\log(X)}}{\sqrt{n}}$ (A11)

Reference

[1] Limpert, E., W. A. Stahel, and M. Abbt. 2001. Log-normal distributions across the sciences: Keys and clues. *Bioscience* 51:341-352.
