# Supplementary material for: NORMA-Gene: A simple and robust method for qPCR normalization based on target gene data
Source: BMC Bioinformatics. 2011 Jun 21;12:250. doi: 10.1186/1471-2105-12-250 (PMC3223928; doi:10.1186/1471-2105-12-250)
Supplement: Additional file 2 — Figure S1. The figure shows scatter of the normalized mean expression values in artificial data (see summary of this statistics in Figure 2). [file 1471-2105-12-250-S2.DOC]

**Additional file 2: Figure S1.**

Figure showing scatter of the normalized mean expression values in artificial data. Each data point represents a single gene within a replicate. Each combination of variance parameters were sampled from eight genes per replicate and in forty replicates (yielding 320 data points). A value of one represents the true mean of each data point. Gray diamonds represent NORMA-Gene normalized raw data, and open triangles represent normalization of raw data to a reference factor (that could represent normalization to either a single reference gene or to a factor based on more reference genes). Each panel represent the distribution of one round of artificial data generation (40 replicates) and three panels are shown for each combination of parameters. Overall, NORMA-Gene normalized artificial data deviates the least from one and produces less extreme values when bias-to-variation ratios decrease and when reference factors are measured equally precise as target genes corroborating the results of normalization of real data (see summary of this statistics in figure 2B&C).

Bias 20, NF 10, Target 10

Bias 15, NF 10, Target 10

Bias 10, NF 10, Target 10

Bias 7.5, NF 10, Target 10

Bias 5, NF 10, Target 10

Bias 10, NF 7.5, Target 10

Bias 10, NF 5, Target 10

Bias 10, NF 2.5, Target 10
