# Supplementary material for: NORMA-Gene: A simple and robust method for qPCR normalization based on target gene data
Source: BMC Bioinformatics. 2011 Jun 21;12:250. doi: 10.1186/1471-2105-12-250 (PMC3223928; doi:10.1186/1471-2105-12-250)
Supplement: Additional file 3 — Table S1-Overview of analyzed data-sets. The table displays a table of experimental information related to the analysed data-sets (I-III). [file 1471-2105-12-250-S3.DOC]

**Additional file 3: Table S1 - Overview of analyzed data-**sets

| **Data-set I (Springtail)** | **Data-set II (Earthworm)** | **Data-set III (*Daphnia*)** |
| --- | --- | --- |
| Test organism: *Megaphorura arctica* | Test organism: *Eisenia fetida* | Test organism: *Daphnia magna* |
| Stressor(s): Cold and drought | Stressor(s): Silver | Stressor(s): Ibuprofen |
| Test duration: 15 days | Test duration: 14 days | Test duration: 48 hrs |
| **Target genes** | **Target genes** | **Target genes** |
| *Aquaporin* | *Catalase* | *Chitinase* |
| *Δ9-Fatty acid desaturase* | *Coelomic cytolytic factor 1* | *Choline-phosphate cytidylyltransferase* |
| *Glutathione peroxidase* | *Heat shock protein 70* | *Cyclooxygenase* |
| *Heat shock protein 70* | *Lysozyme* | *Fatty acid binding protein 3* |
| *Superoxide dismutase (Cu/Zn)* | *Metallothionein* | *Juvenile hormone esterase* |
| *Trehalose-6-phosphate synthase* | *MEK kinase 1* | *Leukotriene B4 12-hydroxydehydrogenase* |
|  | *Myeloid differentiation factor 88* | *Retinoid x receptor* |
|  | *Protein kinase C* | *Triacylglycerol lipase* |
|  | *Superoxide dismutase (Cu/Zn)* | *Vitelline outer layer membrane protein 1* |
|  |  | *Vitellogenin 1* |
|  |  |  |
| **Reference genes** | **Reference genes** | **Reference genes** |
| *Beta-actin* | *Beta-actin* | *Beta-actin* |
| *TATA-box-binding protein* (non-valid) | *Cyclophilin A* (non-valid) | *Glyceraldehyde-3-phosphate dehydrogenase* |
|  | *Pyruvate carboxylase* (non-valid) | *Ubiquitin conjugating enzyme* |
