# Supplementary material for: NORMA-Gene: A simple and robust method for qPCR normalization based on target gene data
Source: BMC Bioinformatics. 2011 Jun 21;12:250. doi: 10.1186/1471-2105-12-250 (PMC3223928; doi:10.1186/1471-2105-12-250)
Supplement: Additional file 4 — Figure S2. The figure provides a validation of the effect of number of genes on mean and standard deviation of the fitted a for the real data-sets. [file 1471-2105-12-250-S4.DOC]

# Additional file 4: Figure S2. Validation of effect of number of genes on mean (left) and standard deviation (right) of fitted *a*. The outcome depends on the particular genes that are included in the analysis, and the sequence by which genes are entered thus makes a difference. Here three random sequences (permutations) were used for each of the three data-sets. Large variation is evident when few genes are used; however, when five or more genes are used the variation is limited and NORMA-Gene provides consistent and valid normalizations.

| **Data-set I** |  |
| --- | --- |
|  |  |
| **Data-set II** |  |
|  |  |
| **Data-set III** |  |
|  |  |
