# Supplementary material for: NORMA-Gene: A simple and robust method for qPCR normalization based on target gene data
Source: BMC Bioinformatics. 2011 Jun 21;12:250. doi: 10.1186/1471-2105-12-250 (PMC3223928; doi:10.1186/1471-2105-12-250)
Supplement: Additional file 5 — Figure S3. The figure shows the relative effect of normalization on expression values in each replicate for the real data-sets. [file 1471-2105-12-250-S5.DOC]

Additional file 5: Figure S3.

The figure shows the relative effect of normalization on variation in each treatment. Each point represents the log_10_ transformed ratio between normalized standard variation (s.d.) divided by the s.d. of raw data. A value of zero represents no change, while negative values signify reduced variance of normalized data compared to raw data. Open circles represent NORMA-Gene normalized raw data, and gray triangles represent normalization of raw data to one reference gene or to a normalization factor based on three genes (data-set III only). (**A**) Data-set I. (**B**) Data-set II. (**C**) Data-set III.

| A | B | C |
| --- | --- | --- |
|  |  |  |
