# Supplementary material for: NORMA-Gene: A simple and robust method for qPCR normalization based on target gene data
Source: BMC Bioinformatics. 2011 Jun 21;12:250. doi: 10.1186/1471-2105-12-250 (PMC3223928; doi:10.1186/1471-2105-12-250)
Supplement: Additional file 6 — Figure S4. The figure shows the relative effect of normalization on variation in each treatment for the real data-sets. [file 1471-2105-12-250-S6.DOC]

# Additional file 6: Figure S4. Scatter of the relative effect of normalization on expression values in each replicate. The entries represent the correction factor for each replicate given as the log_10_ transformed value of normalized relative to raw expression (i.e. a value of zero represents no change to the raw data). For normalization by geNorm (data-set III) and NORMA-Gene a single common correction factor is calculated for each replicate across genes, while there is a slightly deviating effect on different genes of reference gene normalization. Here an average of all genes is given. Gray diamonds represent NORMA-Gene normalized raw data, and open triangles represent normalization of raw data to one reference gene (or normalization to a factor based on three genes, data-set III only). Overall, NORMA-Gene normalized data deviate the least from zero and produce less extreme values. Hence, NORMA-Gene has the lowest effect on the relative mean expression values providing the most robust normalization.

| **Data-set I** | **Data-set II** | **Data-set III** |
| --- | --- | --- |
|  |  |  |
